# Behavioural Characterisation and Pharmacological Validation of an Incisional Wound‐Related Pain Model

**DOI:** 10.1002/ejp.70284

**Published:** 2026-05-08

**Authors:** Catherine R. Healy, Maria C. Redmond, Mary Hopkins, Georgina Gethin, Abhay Pandit, David P. Finn

**Affiliations:** ^1^ Pharmacology and Therapeutics, School of Pharmacy and Medical Sciences, Institute for Health Discovery and Innovation University of Galway Galway City Ireland; ^2^ Galway Neuroscience Centre University of Galway Galway City Ireland; ^3^ Centre for Pain Research University of Galway Galway City Ireland; ^4^ CÚRAM, Research Ireland Centre for Medical Devices University of Galway Galway City Ireland; ^5^ School of Nursing and Midwifery University of Galway Galway City Ireland; ^6^ Alliance for Research and Innovation in Wounds University of Galway Galway City Ireland

## Abstract

**Background:**

Wound‐related pain represents a significant unmet clinical need. To advance its understanding and treatment, there is a need for a validated preclinical model for the study of wound‐related pain in both sexes.

**Methods:**

A 1.2 cm incision was created on the hairy skin of the dorsum of male and female Sprague–Dawley rats posterior to L4. In the first study, mechanical withdrawal thresholds at the dorsum and hind paws were assessed for 34 days post‐incision in both male and female rats to investigate the temporal profile of mechanical hypersensitivity following a dorsum incision. The second study investigated the effects of morphine (3 mg/kg s.c.) on mechanical hypersensitivity in the dorsum and hind paws on post‐incision Day 8 in male Sprague–Dawley rats.

**Results:**

Robust mechanical hypersensitivity was evident in the dorsum up to 14 days post‐incision in males and 7 days post‐incision in females, indicating sex differences in the temporal profile of mechanical hypersensitivity following incision. Secondary mechanical hypersensitivity was present in the hind paws of both sexes following a dorsum incision. Morphine (3 mg/kg s.c.) significantly attenuated dorsum and hind paw mechanical hypersensitivity on post‐incision Day 8.

**Conclusions:**

These results suggest that the dorsum incision model is suitable for modelling incisional wound‐related pain and exhibits sex differences in pain‐related behaviour post‐incision.

**Significance:**

This paper extends the characterisation of an animal model of incisional wound‐related pain, in both sexes. It represents an advancement in the ability to model this condition pre‐clinically and will facilitate investigation of underlying mechanisms and novel therapeutics.

## 1 Introduction

Wound‐related pain is a significant unmet clinical need, affecting approximately 80% of individuals with lived experience of chronic wounds (Woo and Sibbald 2008; Probst et al. 2023). Current wound management strategies typically do not focus on pain as a primary concern (Bechert and Abraham 2009), and patient panels have identified wound pain as a research priority (Probst et al. 2023). Higher levels of acute postoperative wound‐related pain are a risk factor for the development of chronic post‐surgical pain (Rosenberger and Pogatzki‐Zahn 2022). The mechanisms of sensitisation following incision may differ from those of inflammatory or neuropathic pain (Pogatzki‐Zahn et al. 2017). Incisional wound‐related pain is thought to arise primarily from tissue inflammation and peripheral and central sensitisation (Hill et al. 2010). To advance understanding of mechanisms and develop novel therapeutics, a well‐characterised and validated rodent model of wound‐related pain is required.

Current animal models used to study wound‐healing and post‐surgical pain may be suitable for studying wound‐related pain. Models of post‐surgical pain, such as plantar incision, have significantly advanced understanding of the mechanisms of incision‐related pain; however, because of the location of the incision on glabrous skin, this model may not be the most suitable for studying wound‐related pain. To note, cutaneous wound healing processes differ between species, due to factors such as the presence of the panniculus carnosus and differences in skin composition, which is important when considering the study of wound healing or pain‐related behaviour following wounding, particularly when considering the translational elements of the model (Segelcke et al. 2023). Whilst these factors should be considered and are important in interpretation of the results of studies such as those included herein, preclinical rodent models are nevertheless useful to advance our understanding of potential novel therapeutic strategies that could be utilised for under‐studied conditions, such as wound‐related pain. The hairy skin dorsum incision model developed by Duarte et al. (2005) involved the creation of a 1 cm incision on the rat dorsum and evaluation of ‘allodynic‐like’ and ‘hyperalgesia‐like’ behaviours. Further work modified this model to a 1.2 cm incision with the addition of blunt dissection around the wound and demonstrated similar behavioural outcomes (Ohri et al. 2013). To date however, no studies have investigated the dorsum incision model in female animals, or studied potential secondary hypersensitivity (e.g., in the hind paws) in the model, given that research involving the gastrocnemius model has indicated secondary hypersensitivity following incision (Pogatzki et al. 2002). The experiments described herein sought to address these questions while also extending characterisation of the temporal profile of pain‐related behaviour in the model.

Sex differences in wound healing processes are known (Ashcroft and Mills 2002; Ashcroft et al. 1997). Segelcke et al. (2025) identified the need for further studies on sex as a biological variable in post‐surgical pain models, and we aimed to address this research gap in the context of incisional wound‐related pain. In addition, preclinical models must model the clinical features of the condition, with similar aetiologies and underlying processes, and respond to treatments (e.g., opioids) that show efficacy in the clinical wound pain or post‐surgical pain population, ensuring robust face, construct and predictive validity (Segelcke et al. 2025). While previous work has investigated the effect of morphine on post‐incisional primary hypersensitivity (Duarte et al. 2005), the second experiment described herein aimed to investigate morphine's effects at a later timepoint when both primary and secondary mechanical hypersensitivity were robustly evident.

The aim of the first experiment described herein was to characterise the time course of pain‐related behaviour following a dorsum incision in rats of both sexes, both at the level of the incision (dorsum) and in the hind paws. The second experiment investigated the effect of morphine on mechanical hypersensitivity following dorsum incision to further establish the predictive validity of the model.

## 2 Methods

### 2.1 Animals

Thirty‐six Sprague–Dawley rats (18 males and 18 female, 6–7 weeks, 130–180 g on arrival for females, 150–200 g on arrival for males; Charles River, UK) were used in Experiment 1. Thirty‐six male Sprague–Dawley rats (6–7 weeks, 160–200 g on arrival; Charles River UK) were used in Experiment 2. Rats were maintained under a standard 12:12 h light/dark cycle (lights on from 07:00–19:00 h) in a temperature (21°C ± 2°C) and humidity‐controlled (45%–55%) room throughout the study. The light intensity in the room was approximately 200 lx and approximately 30 lx at the cage floor level. Food (14% Harlan Teklad 2014 Maintenance Diet, Envigo, UK) and water were provided ad libitum. All behavioural procedures were performed in the morning during the light phase. Rats were housed in clear polycarbonate cages (45 × 20 × 20 cm^3^; 800 cm^2^ floor area) containing ABP3 poplar bedding (Datesand Ltd., United Kingdom), Sizzle‐Pet material (LBS Biotechnology, Horley, UK), and a red plastic tunnel for environmental enrichment. All rats were pair‐housed and acclimatised for at least five days prior to single housing.

All experimental procedures were approved by the Animal Care and Research Ethics Committee of the University of Galway. The study was conducted under a Project Authoriation from the Health Products Regulatory Authority in the Republic of Ireland and in accordance with the EU Directive 2010/63. This study was reported in accordance with the ARRIVE 2.0 guidelines (Percie Du Sert et al. 2020).

### 2.2 Experimental Design

#### 2.2.1 Experiment 1

**FIGURE 1:**
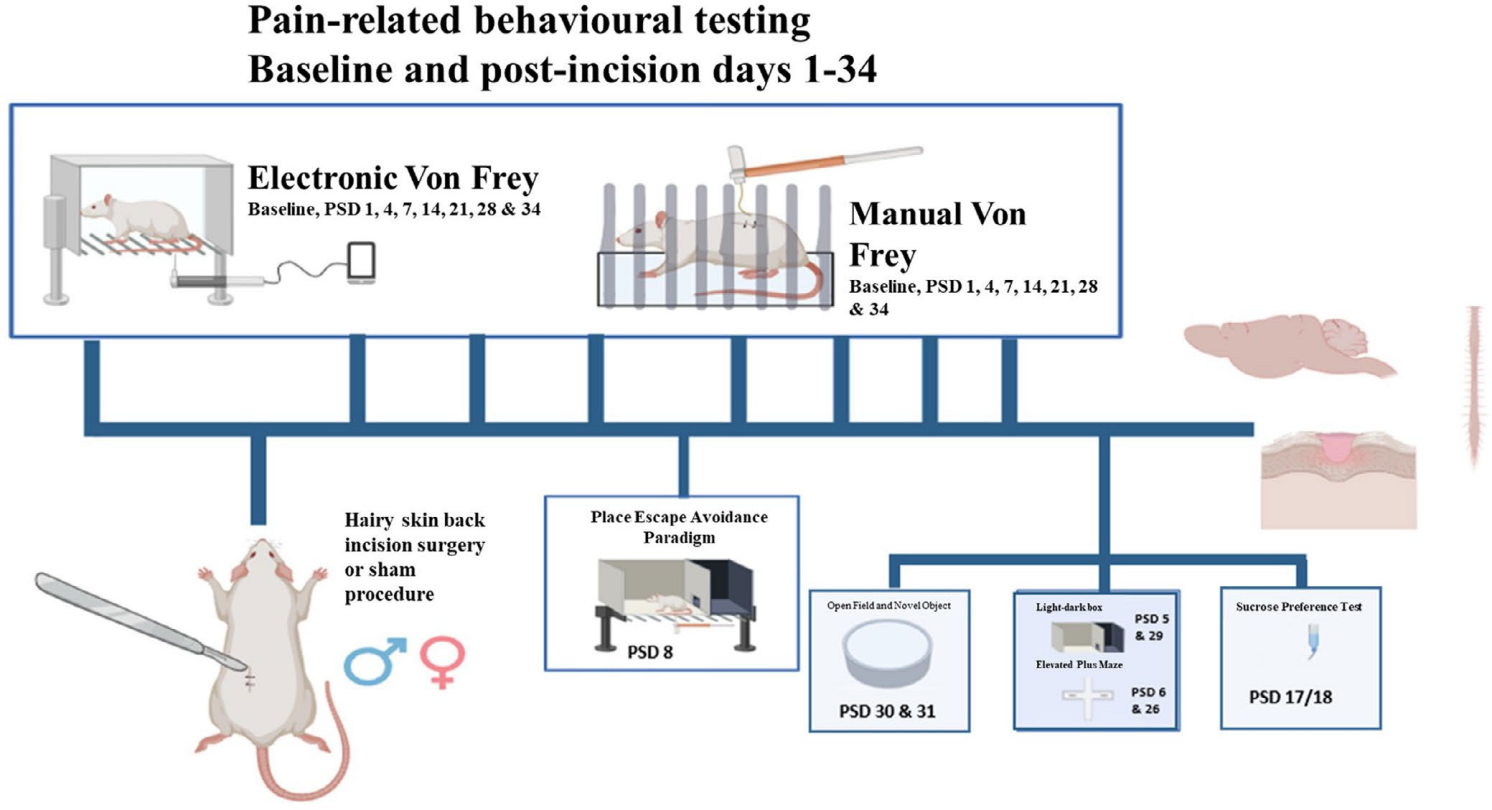
Experimental timeline for hairy skin back incision characterisation study (Experiment 1). The diagram outlines the timeline over which different behavioural tests were performed to assess pain‐related behaviour. PSD, days post‐incision or sham procedure day.

The experimental design is shown in Figure 1. Upon single housing, rats underwent a further 5 days of habituation prior to shaving of the dorsum under brief isoflurane anaesthesia to facilitate behavioural testing. Thereafter, the rats were individually housed for the duration of the experiment. 24 h after shaving the dorsum, baseline mechanical withdrawal threshold responses of the dorsum or hind paws were obtained for each rat using the manual Von Frey or electronic Von Frey, respectively. Two baseline tests were conducted for each paradigm to ensure a stable baseline. Baseline mechanical withdrawal thresholds were determined 7 and 4 days prior to the dorsum incision or sham procedure. After baseline testing, rats of each sex were pseudo‐randomly assigned to either the sham or incision group (*n* = 9 rats per group per sex), ensuring no significant differences in the average body weight or baseline mechanical thresholds between the different experimental groups. The experimental group allocation was performed by another researcher. The dorsum of each rat was re‐shaved (without anaesthesia) 24 h prior to each post‐incision/sham pain‐related behavioural testing session. Mechanical withdrawal thresholds were obtained on Days 1, 4, 7, 14, 21, 28 and 34 post‐incision/sham procedure to assess the development and temporal profile of mechanical hyper(sensitivity). Rats were euthanised via live decapitation on Day 35 post‐incision/sham procedure. All experimental procedures were performed by a female researcher.

#### 2.2.2 Experiment 2

**FIGURE 2:**
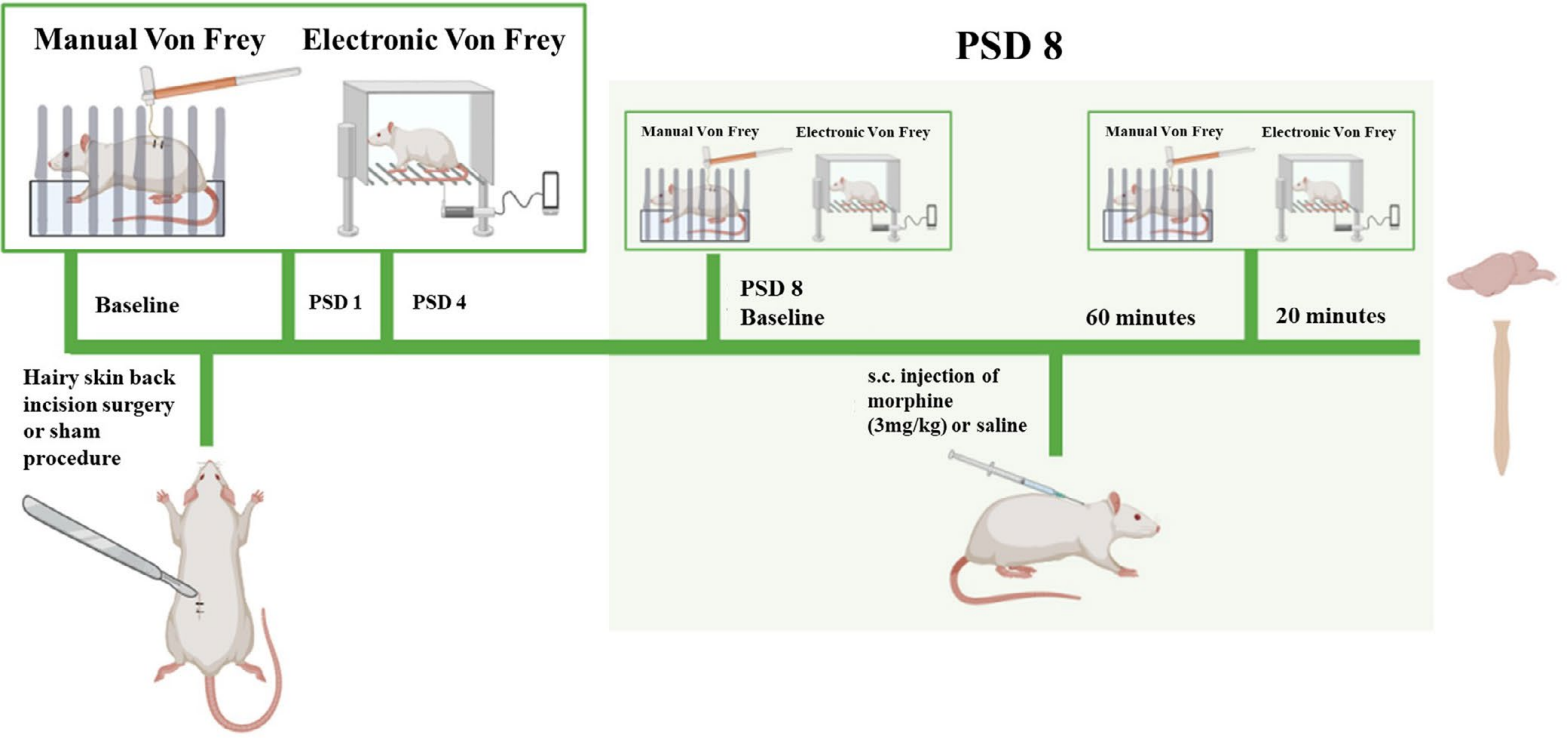
Schematic outline of the experimental timeline for investigating the effect of morphine (3 mg/kg) or saline administration on pain‐related behaviour following incisional wound (Experiment 2).

The experimental design is shown in Figure 2. The rat dorsum was shaved under isoflurane anaesthesia four days after single housing to facilitate behavioural testing. Baseline dorsum and hind paw mechanical withdrawal thresholds were obtained 4 and 2 days prior to the incision or sham procedure. After baseline testing, the rats were pseudo‐randomly assigned to either the sham or incision group (*n* = 18 per group), ensuring no significant differences in the average body weight or baseline mechanical withdrawal thresholds between the groups. Following pain‐related behavioural testing on Day 4 post‐incision/sham procedure, rats were pseudo‐randomly assigned to receive morphine or vehicle on Day 8 post‐incision/sham procedure, ensuring no significant differences in pre‐morphine/vehicle mechanical withdrawal thresholds between groups. There were four experimental groups: Sham + Saline (*n* = 9), Sham + Morphine (*n* = 9), Incision + Saline (*n* = 9) and Incision + Morphine (*n* = 9). Experimental group allocation was carried out by another researcher. On post‐incision/sham Days 1, 4 and 8, manual Von Frey testing was performed on the dorsum, followed by hind paw electronic Von Frey testing. Following behavioural testing on the morning of post‐incision/sham Day 8, rats were returned to the home cage and subsequently received a subcutaneous (s.c. 1 mL/kg) injection of sterile saline (0.89% *w*/*v* NaCl) or morphine sulphate (3 mg/kg), dissolved in sterile saline. Rats were returned to the home cage for 40 min post‐injection and subsequently re‐habituated in the behavioural testing arenas until mechanical withdrawal threshold testing at 60 min post‐drug administration. The time point for behavioural testing post‐morphine administration was based on published studies showing the efficacy of morphine in reducing pain‐related behaviours in rodents 60 min post‐administration (Hestehave et al. 2019; Whiteside et al. 2004). Rats were euthanised via live decapitation 80 min after drug administration, immediately post‐behavioural testing. The researcher who performed the behavioural testing was female and was blinded to group allocation and drug treatment.

### 2.3 Shaving and Marking of the Dorsum

**FIGURE 3:**
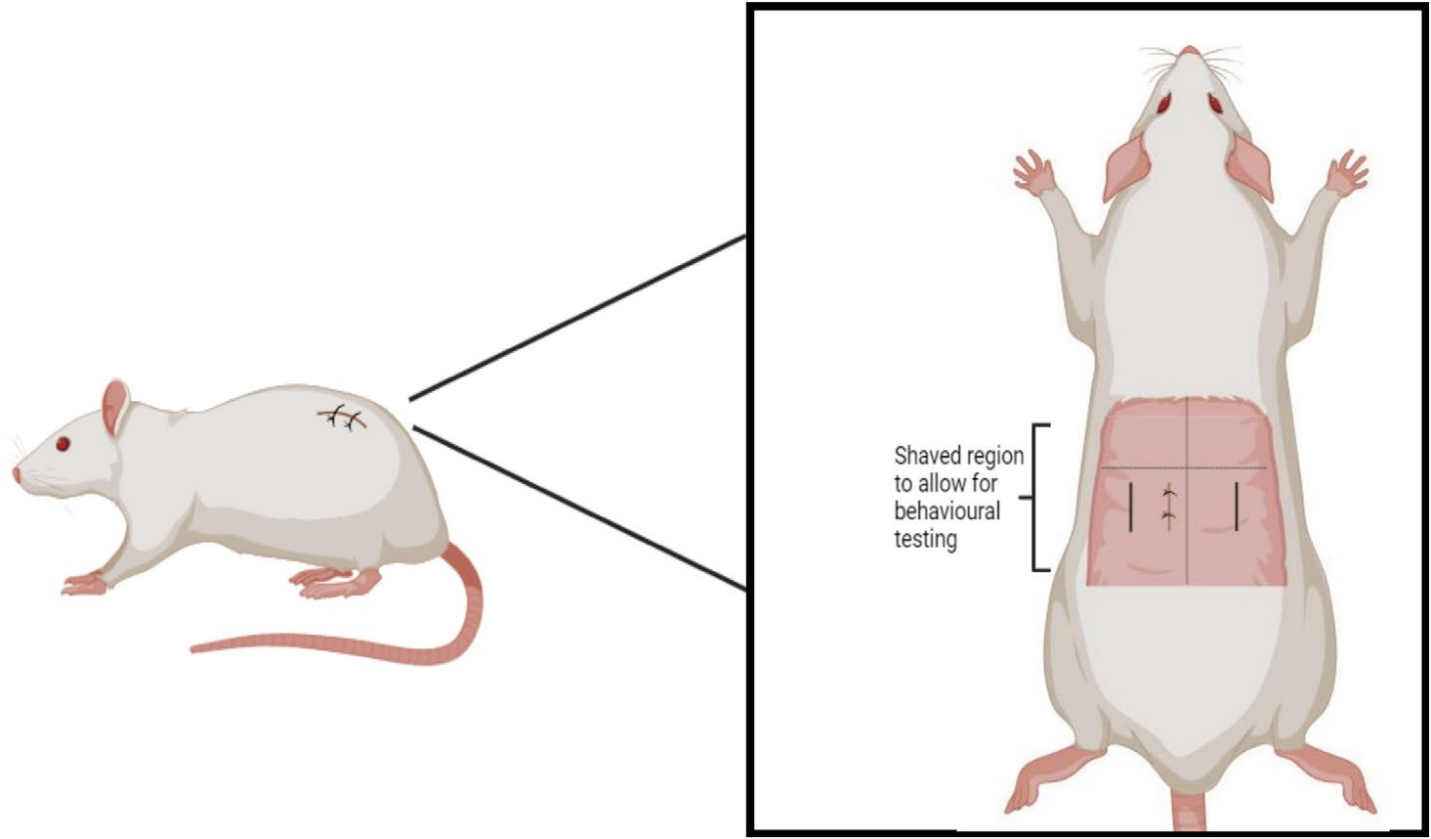
Shaving and marking of the rat dorsum for incisional wound creation and behavioural testing. Measurements for marking the dorsum and location of the wound are described in the Methods section. The wound was created on the left of the midline, with parallel marks created 1 cm ipsilateral and 2 cm contralateral to the wound for behavioural testing. Both marks for behavioural testing were 1.5 cm from the midline of the rat and 1.2 cm in length.

Shaving and marking of the dorsum were completed under isoflurane anaesthesia 24 h before the first baseline test (Figure 3). A pre‐procedure evaluation was performed for each rat, including body weight check, prior to anaesthesia. Rats were initially anaesthetised in a Plexiglass induction chamber with 5% isoflurane (Fisher Scientific, UK) in 0.8 L/min O_2_. The depth of anaesthesia was determined by the loss of responsiveness to tail pinch. Anaesthesia was maintained using 1.5%–2.5% isoflurane in 0.8 L/min O_2_, and rats were monitored constantly for the duration of the procedure, with checks for body temperature, tail reflex and respiratory rate performed every 5 min. Under anaesthesia, the dorsum was shaved from the lower thoracic region to the top of the hipbone line using an electronic shaver. The midline was marked using a fine‐tipped, non‐toxic permanent marker. The length of the dorsum was measured using a flexible plastic ruler (Fine Science Tools, Heidelberg, Germany). A horizontal line at the level of the L4 transverse process was created using the following method: 1. The length of the rat was 15 cm from the base of the ear (posterior) to the base of the tail: a horizontal line was created at 8 cm across. 2. The length of the rat was 14 cm from the base of the ear (posterior) to the base of the tail: a horizontal line was created at 7.5 cm across. 3. The length of the rat was 13 cm from the base of the ear (posterior) to the base of the tail, and a horizontal line was created at 7 cm across. These reference points were validated by bringing both forefingers together across the bottom of the ribcage to meet over the spine, corresponding to the L1 vertebra. The experimenter counted four vertebrae to validate the reference mark made at L4. A right‐angled ruler was used to mark 0.5 cm to the left of the midline and 0.5 cm down from the L4 transverse process. A 1.2 cm line was marked vertically from this point (to mark the incision line) in the posterior direction and parallel to the spine. Behavioural testing sites were marked on the dorsum by creating a line 1 cm ipsilateral (left) and 2 cm contralateral (to the right) of the incision line. Figure 3 depicts the shaving and marking locations. The average duration of anaesthesia for this procedure was 5 min. Following recovery from anaesthesia, the rats were placed individually into a recovery chamber maintained at 26°C for 30 min before being returned to the home cage. To facilitate brief repeated shaving and reinforcement of the lines for behavioural testing on the dorsum in awake behaving rats throughout the experiment, the rats were habituated to the electronic shaver throughout the habituation period to minimise stress.

### 2.4 Rat Back Hairy Skin Incision Procedure, or Sham

The incision procedure was performed as previously described (Duarte et al. 2005; Ohri et al. 2013). Pre‐surgical evaluation of body weight was performed before the induction of anaesthesia. Anaesthesia was induced and maintained as described above for the shaving and marking procedures. The depth of anaesthesia was evaluated by the loss of responsiveness to tail pinch. Respiratory rate, body temperature and withdrawal reflex were assessed every 5 min for the duration of the procedure. Under anaesthesia, the shaved area of the dorsum was re‐shaved and disinfected with chlorhexidine solution to ensure aseptic conditions. Vidisic (0.2% *w*/*w*) eye gel (Bausch&Lomb UK Ltd., UK) was placed in the eyes to maintain moisture. The dorsum was re‐marked under aseptic conditions. A 1.2 cm incision was made using a no. 10 surgical blade attached to a blade holder 0.5 cm posterior to the L4 transverse process and 0.5 cm to the left of the midline. Blunt dissection of the skin away from the underlying fascia was performed using blunt scissors (Fine Science Tools, Germany) at a radius of 0.5 cm around the wound. The area was cleaned with sterile saline and cotton buds. The wound was closed using two non‐absorbable 4‐0 Mersilk sutures (Johnson and Johnson, Ireland). Great care was taken to ensure that the wound margins were gently brought together directly opposing each other. For the sham procedure, rats were exposed to the same anaesthetic conditions and marking of the dorsum; however, incision, blunt dissection and suturing were not performed. The duration of each sham/incision procedure was 10–15 min from the time of anaesthesia induction. After the incision or sham procedure, the rats were placed individually in a recovery chamber maintained at 26°C for a minimum of 30 min before returning to the home cage. General health and well‐being were monitored twice in the first 24 h post‐surgery and then daily thereafter for the duration of the experiment.

### 2.5 Pain‐Related Behavioural Testing

Pain‐related behavioural testing on the dorsum and hind paws was completed in custom‐designed stainless steel individual arenas placed on top of a wire mesh base. These custom arenas facilitated the testing of both the dorsum and hind paws without the need to change the arenas. Arenas were built by Custom Stainless, Ireland, based on specifications (240 × 100 × 100 cm^3^) provided following trial work using training rats. A clear Perspex ‘door’ prevented the rats from exiting.

### 2.6 Manual Von Frey

Manual Von Frey assessed mechanical (hyper)sensitivity at 1 cm ipsilateral and 2 cm contralateral to the incision on the dorsum, using nylon Von Frey filaments (Touch Test Sensory Evaluator #58011, Stoelting, USA) ranging from 0.6 g to 15 g. A maximum filament weight of 15 g was used to prevent any potential local irritation (Ohri et al. 2013). Lux at the testing arena level was 40 lx. Testing was completed at approximately the same time of day in a quiet, temperature‐controlled room.

The rats were habituated for 30 min prior to the initial exposure to the arena. An additional 5 min of habituation was provided upon the entry of the experimenter into the behavioural testing room. Rats were first stimulated at the 2 cm contralateral testing site with the lowest weight filament (0.6 g post‐incision/sham, 2 g for baseline testing). The Von Frey filament was pressed against the skin until it bent four times per filament at a frequency of approximately once every 5 s. The response of each filament was recorded. A positive response included licking or biting at the site of application, shaking, shuddering, rapid turn and vocalisation or robust contraction of the skin and subdermal muscles under the testing location, known as the cutaneous trunk muscle response (CTMr). Following the application of the lowest weight filament to the 2 cm contralateral testing site, the 2 cm contralateral testing site of rats in adjacent and subsequent arenas was tested using the same filament. Following the application of the lowest weight filament to the 2 cm contralateral testing site of all rats in the arena, the 1 cm ipsilateral testing site of each rat was stimulated with the same filament weight. This was repeated for filaments of ascending weights until the mechanical response threshold was reached for each testing site. Rats were assigned a mechanical withdrawal threshold of 15.1 g if they did not respond 4/4 times to the maximum filament weight of 15 g.

### 2.7 Electronic Von Frey

The electronic Von Frey test assessed mechanical (hyper)sensitivity in the left and right hind paws pre‐ and post‐incision or sham procedure. The electronic Von Frey test was performed 5 min after the completion of the manual Von Frey test under the same experimental testing conditions. The 5 min gap between mechanical modalities allowed for any potential confounding effects of manual Von Frey testing on electronic Von Frey testing via diffuse noxious inhibitory control of pain (DNIC) to subside. For DNIC induced by mechanical stimulation (tail or muzzle pinch), a mean inhibitory period of 62 s prior to tail pinch recovery has been previously reported (Dickenson et al. 1981).

An electronic Von Frey anesthesiometer (IITC Life Science Inc., USA) was used to assess hind paw mechanical hypersensitivity. The IITC aesthesiometer used 0.8 mm rigid, non‐hygroscopic polypropylene tips. To ensure accuracy, a standardised weight of 7.00 g was placed on the cone of the probe prior to testing. Three applications were used to evaluate the mechanical sensitivity of each paw. Alternating between the left (ipsilateral) and right (contralateral) paws allowed for at least a 5‐min interval between paws. The method of electronic Von Frey testing was based on the test protocols routinely used in our laboratory and others (Haranishi et al. 2021; Mulpuri et al. 2018; Wen et al. 2024; Di Marino et al. 2025; Boullon et al. 2025). Starting with the right paw, a filament was applied perpendicular to the plantar surface of the hind paw with a continuous and steady force until a positive withdrawal response was observed. A positive result was recorded if flinching, licking, or paw withdrawal occurred in response to filament application. The applied force (grams) was recorded and considered as the mechanical withdrawal response for that trial. The withdrawal threshold of the right hind paw of rats was tested in the subsequent arenas. Following the completion of the first trial on the right paw of each rat, the left paw was stimulated in the same manner. This procedure was repeated twice more, commencing with the right paw, followed by stimulation of the left paw until three readings were recorded for each paw. The mechanical withdrawal threshold for each paw was calculated by taking the average response (grams) across three trials per paw.

### 2.8 Chemicals and Drug Preparation

The mu‐opioid receptor agonist morphine (morphine sulphate, 10 mg/mL, solution for injection; Mercury Pharma International Ltd., Ireland) was purchased from a local pharmacy in Galway, Ireland, under a controlled drug licence from the Health Products Regulatory Authority. Morphine sulphate (provided in a glass ampoule as a 10 mg/mL solution) was dissolved in sterile saline (0.89% *w*/*v* NaCl) to a concentration of 3 mg/mL and administered subcutaneously (s.c.) in an injection volume of 1 mL/kg (3 mg/kg). Correction for the free base versus salt form of morphine sulphate was not performed. Sterile saline (0.89% *w*/*v* NaCl) was administered as a vehicle in an injection volume of 1 mL/kg. The morphine solution was freshly prepared on the day of the experiment.

### 2.9 Statistical Analysis

SPSS statistical software (IBM SPSS Statistics, version 27 for Windows; SPSS Inc., USA) was used for the statistical analysis. Normality and homogeneity of variance were assessed using the Shapiro–Wilk and Levene's tests, respectively.

For experiment 1, parametric time course behavioural data were analysed using two‐way repeated measures analysis of variance (RM ANOVA) with sex (male or female) and procedure (incision or sham) as the between‐subject factors, and time as the within‐subject factor for experiment 1, with post hoc Tukey's Honest Significant Difference where applicable. For experiment 2, parametric time course behavioural data were analysed via one‐way RM ANOVA with procedure (sham or incision) as the between‐subject factor, and time as the within‐subject factor with post hoc independent *t*‐test with Bonferroni–Holm correction. For RM ANOVA, the sphericity of the datasets was ascertained by Mauchly's Test for Sphericity; if the assumption for sphericity was violated, Greenhouse–Geisser correction was used.

For experiment 1, non‐time course parametric data were analysed using two‐way ANOVA, with between‐subject factors of sex (male or female) and procedure (incision or sham). For experiment 2, non‐time course parametric data were analysed using two‐way ANOVA, with procedure (sham or incision) and drug (vehicle or morphine) as the between‐subject factors. Post hoc analysis was performed using Tukey's Honest Significant Difference test, where appropriate. A paired‐samples *t*‐test with Bonferroni–Holm correction was used on parametric data to assess within‐subject pairwise comparisons to baseline in both experiments.

Outliers were determined based on the data distribution. If a data point fell out of the range of (mean−2 × standard deviation) to (mean+2 × standard deviation), it was considered an outlier and excluded from the analysis. If the data were not normally distributed and/or the variance was not homogeneous, transformations were applied. Parametric statistical analyses were then performed on the transformed data. If the data failed normality or homogeneity of variance testing following transformation, non‐parametric statistical analysis was used. Parametric statistical approaches were used if the highest standard deviation of the dataset being analysed was less than or equal to two times the smallest standard deviation (Moore et al. 2009).

Non‐parametric time course data were analysed using Friedman's test with post hoc Mann–Whitney *U* test, with Bonferroni–Holm correction for between‐group pairwise comparisons, or Wilcoxon signed rank test with Bonferroni–Holm correction for within‐subject pairwise comparisons where appropriate. If the data were ordinal/scale or not normally distributed and/or the variance was not homogenous post‐transformation, non‐parametric statistical analysis was performed using the Kruskal–Wallis (KW) one‐way analysis of variance by rank, followed by post hoc Mann–Whitney *U* test, with Bonferroni‐Holm correction.

Data are presented as either ± standard error of the mean (S.E.M) or median with interquartile range (IQR), depending on the statistical approach undertaken, parametric or non‐parametric. All graphs were created using GraphPad Prism software version 8.0 (GraphPad Software, USA). The significance level was *p* < 0.05.

## 3 Results

### 3.1 Dorsum Incisional Wound Creation Leads to Dorsum and Hind Paw Mechanical Hypersensitivity in Male and Female Rats

#### 3.1.1 Experiment 1

In experiment 1, the temporal profile of mechanical sensitivity following a dorsum incision was assessed for 35 days post‐incision.

**FIGURE 4:**
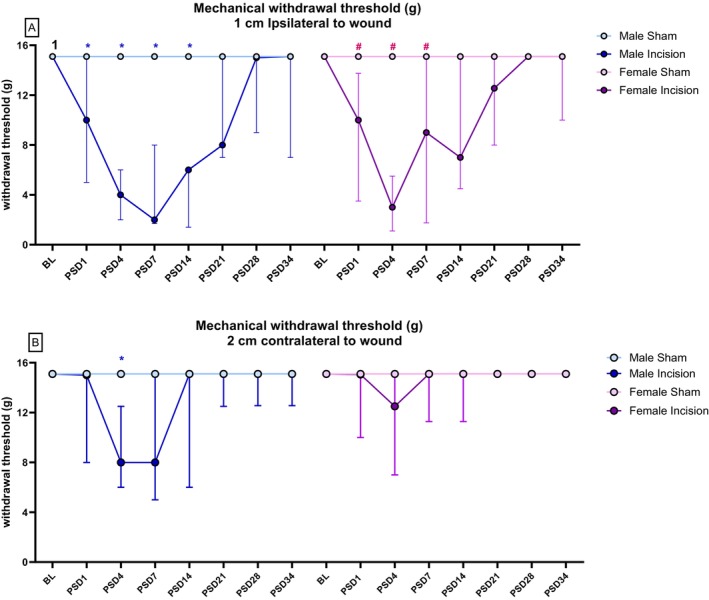
Effect of dorsum incision on mechanical withdrawal thresholds at (A) 1 cm ipsilateral to the wound and (B) 2 cm contralateral to the wound. Data are expressed as median ± interquartile range, *n* = 8/9 per group. Data were analysed using Friedman's test, followed by the Mann–Whitney *U* test with Bonferroni–Holm correction, where appropriate. Data were analysed together but are displayed separately to allow for full visualisation of the data. * *p* < 0.05, Male Sham versus Male Incision. # *p* < 0.05, Female Sham versus Female Incision. ^1^ *p* < 0.007 Male Incision baseline versus PSD 4, 14. Male and female groups were analysed together but are displayed separately for clarity.

Friedman's test revealed significant differences in the mechanical withdrawal threshold at 1 cm ipsilateral to the wound: *χ* ^2^(7) = 52.78 (*p* < 0.01). Mann–Whitney *U* test, followed by Bonferroni–Holm correction, revealed a significantly lower mechanical withdrawal threshold at 1 cm ipsilateral to the wound in male incised rats than in male sham rats on post‐surgery day (PSD) 1, 4, 7 and 14 (*p* < 0.05) (Figure 4A). Mann–Whitney *U* test with Bonferroni–Holm correction revealed a significantly lower mechanical withdrawal threshold at 1 cm ipsilateral to the wound in the female incision versus female sham at PSD 1, 4 and 7 (*p* < 0.05) (Figure 4A). Wilcoxon's test with Bonferroni–Holm correction revealed a significantly lower mechanical withdrawal threshold at 1 cm ipsilateral to the wound in male incision rats at PSD 4 and 14 compared to their baseline (*p* < 0.007). After correcting for multiple comparisons, there was no significant difference in the mechanical withdrawal threshold at 1 cm ipsilateral to the wound in female incision rats compared to baseline.

Friedman's test revealed significant differences in mechanical withdrawal threshold at 2 cm contralateral to the wound: *χ* ^2^(7) = 41.85 (*p* < 0.007). Mann–Whitney *U* test, with Bonferroni–Holm correction revealed significantly lower mechanical withdrawal threshold at 2 cm contralateral to the wound in male incision versus male sham on PSD 4 (*p* = 0.001) (Figure 4B). After correcting for multiple comparisons, there was no significant difference in mechanical withdrawal threshold at 2 cm contralateral for any relevant pairwise comparison compared to baseline.

**FIGURE 5:**
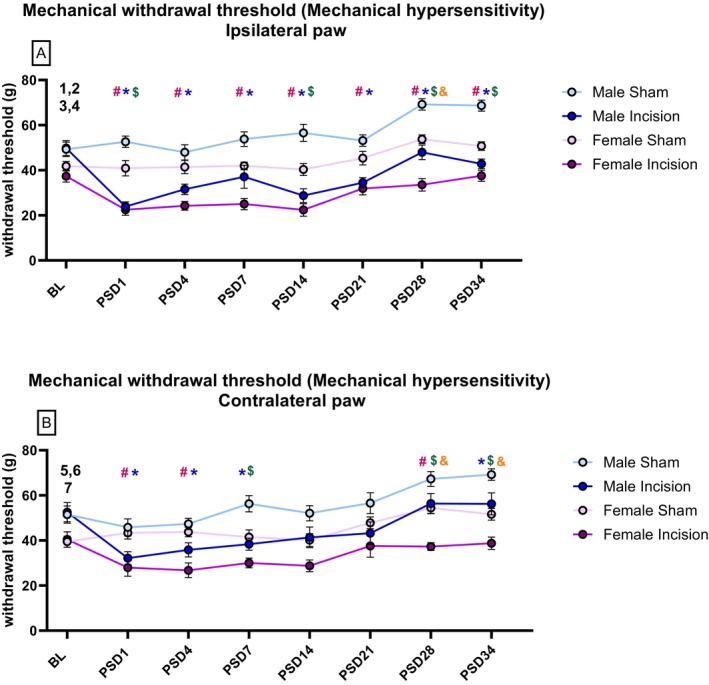
Effect of dorsum incision on paw withdrawal thresholds in the ipsilateral (left) paw (A) contralateral (right) paw (B). Data are expressed as mean ± S.E.M, *n* = 8/9 per group. * *p* < 0.05, Male Sham versus Male Incision. # *p* < 0.05, Female Sham versus Female Incision. $ *p* < 0.05 Female Sham versus Male Sham. & *p* < 0.05, Female Incision versus Male Incision. ^1^ *p* < 0.007 Male Incision baseline versus PSD 1, 4, 14, 21. ^2^ *p* < 0.007 Female Incision baseline versus PSD 1. ^3^ *p* < 0.007 Male Sham baseline versus PSD 28 and 34. ^4^ *p* < 0.007 Female Sham baseline versus PSD 28, 34. ^5^ *p* < 0.007 Male Incision baseline versus PSD 1. ^6^ *p* < 0.007 Male Sham baseline versus PSD 28, 34. ^7^ *p* < 0.007 Female Sham baseline versus PSD 28, 34.

Two‐way repeated measures (RM) ANOVA revealed main effects of time (F_(7,217)_ = 20.67, *p* < 0.05), time × procedure (F_(7,217)_ = 6.91, *p* < 0.05), sex (F_(1,31)_ = 49.93, *p* < 0.05) and procedure (F_(1,31)_ = 142.58, *p* < 0.05) on ipsilateral (left) paw withdrawal threshold (Figure 5A). Tukey's post hoc test revealed significantly lower ipsilateral paw withdrawal threshold in male incision rats versus male sham rats at PSD 1, 4, 7, 14, 21, 28 and 34 (*p* < 0.05) and female incision rats versus female sham at PSD 1, 4, 7, 14, 21, 28 and 34 (*p* < 0.05). Tukey's post hoc test revealed significantly lower ipsilateral paw withdrawal threshold in female sham versus male sham at 1, 14, 28 and 34 (*p* < 0.05). Tukey's post hoc test revealed significantly lower ipsilateral paw withdrawal threshold in female incision versus male incision at PSD 28 (*p* < 0.05) (Figure 5A). Paired sample *t*‐test with Bonferroni–Holm correction revealed significantly lower ipsilateral paw withdrawal threshold in (1) male incision rats at PSD 1, 4, 14 and 21 versus baseline (*p* < 0.007) and (2) female incision rats on PSD 1 versus baseline (*p* < 0.007). Paired sample *t*‐test followed by Bonferroni–Holm correction revealed significantly higher ipsilateral paw withdrawal threshold in (1) male sham rats at PSD 28 and 34 versus baseline (*p* < 0.007) and (2) female sham rats at PSD 28 and 34 versus baseline (*p* < 0.007).

Two‐way RM ANOVA revealed a significant effect of time (F_(7,217)_ = 17.70, *p* < 0.05), sex (F_(1,31)_ = 28.85, *p* < 0.05), procedure (F_(1,31)_ = 26.954, *p* < 0.05), time × procedure (F_(7,217)_ = 5.771, *p* < 0.05) and time × sex (F_(7,217)_ = 2.71, *p* < 0.05), on contralateral (right) paw withdrawal threshold (Figure 5B). Tukey's post hoc test revealed significantly lower contralateral paw withdrawal threshold in male incision versus male sham at PSD 1, 4, 7 and 34 (*p* < 0.05) and in female incision versus female sham at PSD 1, 4 and 28 (*p* < 0.05). Tukey's post hoc test revealed significantly lower contralateral paw withdrawal threshold in female sham versus male sham at PSD 7, 28 and 34 (*p* < 0.05) and female incision versus male incision at PSD 28 and 34 (*p* < 0.05) (Figure 5B). Paired sample *t*‐test followed by Bonferroni–Holm correction revealed significantly lower contralateral paw withdrawal threshold in male incision at PSD 1 versus baseline (*p* < 0.007). After correcting for multiple comparisons, there was no significant difference in contralateral paw withdrawal threshold in female incision rats versus baseline (*p* > 0.007). Paired sample *t*‐test followed by Bonferroni–Holm correction revealed significantly higher contralateral paw withdrawal threshold in male sham rats at PSD 28 and 34 versus baseline (*p* < 0.007) and in female sham rats at PSD 21, 28 and 34 versus baseline (*p* < 0.007).

#### 3.1.2 Experiment 2

In experiment 2, pre‐drug mechanical withdrawal threshold testing was completed to confirm the development of mechanical hypersensitivity in incision rats prior to morphine administration.

**FIGURE 6:**
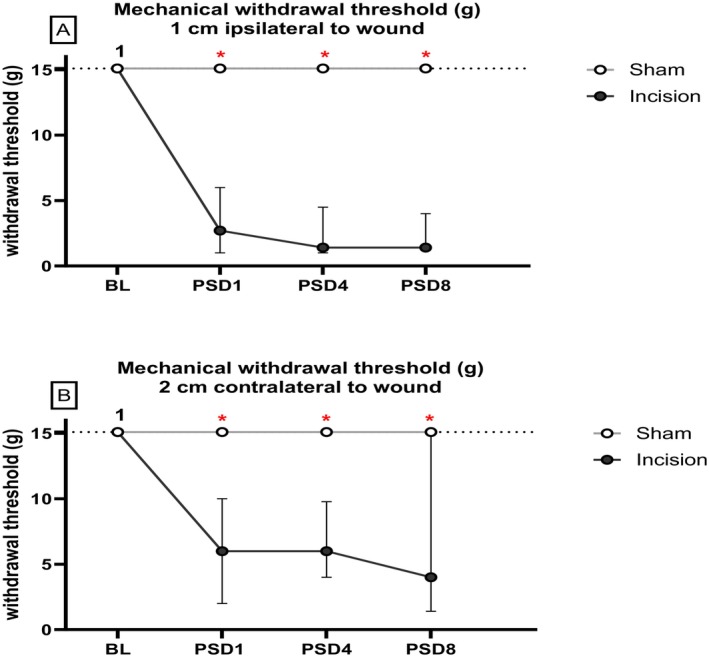
Effect of dorsum incision on mechanical withdrawal thresholds at (A) 1 cm ipsilateral and (B) 2 cm contralateral to the wound site. Data are expressed as median ± interquartile range, *n* = 18 per group. Data were analysed using Friedman's test, followed by the Mann–Whitney *U* test or Wilcoxon test, both with Bonferroni–Holm correction, where appropriate. * *p* < 0.01 Sham versus Incision. ^1^ *p* < 0.0167 Male Incision baseline versus PSD 1, 4 and 8 (pre‐drug).

Friedman's test revealed significant differences in mechanical withdrawal threshold at 1 cm ipsilateral to the wound *χ* ^2^(3) = 37.46 (*p* < 0.0001) on the data prior to drug administration on PSD 8. Mann–Whitney *U* test with Bonferroni–Holm correction revealed a significantly lower mechanical withdrawal threshold at 1 cm ipsilateral to the wound in incision versus sham rats at PSD 1, PSD 4 and PSD 8 before drug administration (*p* < 0.01) (Figure 6A). Wilcoxon's test with Bonferroni–Holm correction revealed significantly lower mechanical withdrawal threshold at 1 cm ipsilateral to the wound in incision rats versus baseline on PSD 1, 4 and PSD 8 pre‐drug (*p* < 0.0167), with no differences for sham.

Friedman's test revealed significant differences in mechanical withdrawal threshold at 2 cm contralateral to the wound *χ* ^2^(3) = 24.91 (*p* < 0.0001) on the data prior to drug administration on PSD 8. Mann–Whitney *U* test with Bonferroni–Holm correction revealed a significantly lower mechanical withdrawal threshold at 2 cm contralateral to the wound in incision versus sham rats at PSD 1, PSD 4 and PSD 8 before drug administration (*p* < 0.01) (Figure 6B). Wilcoxon's test with Bonferroni–Holm correction revealed significantly lower mechanical withdrawal threshold at 2 cm contralateral to the wound in incision rats versus baseline on PSD 1, 4 and PSD 8 pre‐drug (*p* < 0.0167), with no differences for sham.

**FIGURE 7:**
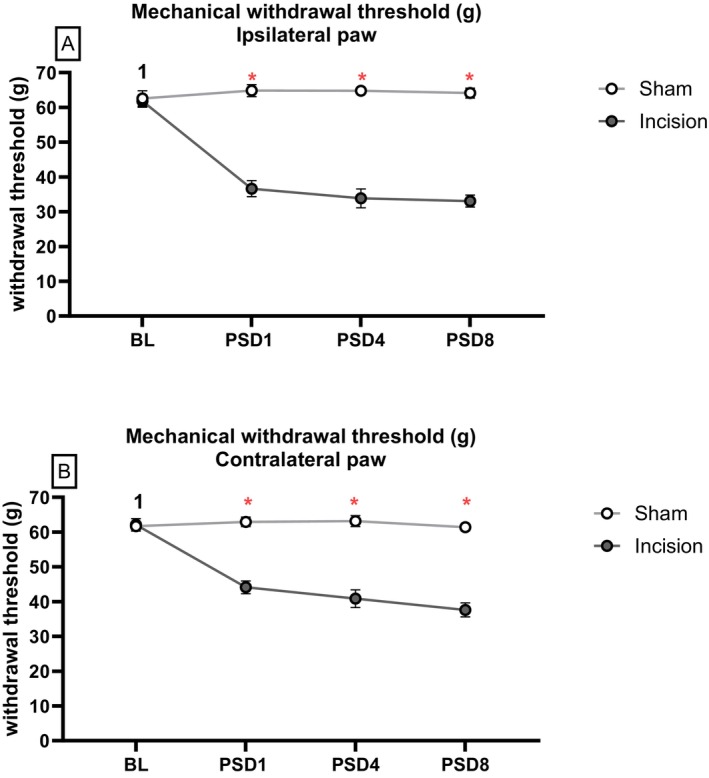
Effect of dorsum incision on mechanical withdrawal thresholds in the (A) ipsilateral (left) and (B) contralateral (right) hind paws. Data are expressed as mean ± S.E.M, *n* = 18 per group. Data were analysed using one‐way RM ANOVA, followed by independent samples *t*‐test with Bonferroni–Holm correction. * *p* < 0.01 Sham versus Incision. ^1^ *p* < 0.0167 Male Incision baseline versus PSD 1, 4 and 8 (pre‐drug).

One‐way RM ANOVA on the data prior to drug administration on PSD 8 revealed a significant effect of time (F_(4,136)_ = 23.45, *p* < 0.05), procedure (F_(1,34)_ = 144.11, *p* < 0.05) and time × procedure (F_(4,136)_ = 33.905, *p* < 0.05) on ipsilateral (left) hind paw withdrawal threshold following dorsum incision. Independent samples *t*‐test with Bonferroni–Holm correction revealed a significantly lower ipsilateral paw withdrawal threshold in incision rats versus sham rats at PSD 1, PSD 4 and PSD 8 before drug administration (*p* < 0.01) (Figure 7A). Paired sample *t*‐test with Bonferroni–Holm correction revealed significantly lower ipsilateral paw withdrawal threshold in incision rats versus baseline at PSD 1,4 and PSD 8 pre‐drug (*p* < 0.0167), with no difference for male sham.

One‐way RM ANOVA on the data prior to drug administration of PSD 8 revealed a significant effect of time (F_(4,136)_ = 29.12, *p* < 0.05), procedure (F_(1,34)_ = 77.09, *p* < 0.05) and time × procedure (F_(4,136)_ = 27.58, *p* < 0.05) on contralateral (right) hind paw withdrawal threshold following dorsum incision. Independent samples *t*‐test with Bonferroni–Holm correction revealed a significantly lower contralateral paw withdrawal threshold in incision rats versus sham rats at PSD 1, PSD 4 and PSD 8 before drug administration (*p* < 0.01) (Figure 7B). Paired sample *t*‐test with Bonferroni–Holm correction revealed a significantly lower contralateral paw withdrawal threshold in incision rats versus baseline at PSD 1,4 and PSD 8 pre‐drug (*p* < 0.0167) with no difference for male sham.

Results from these investigations show the development of robust mechanical hypersensitivity in the dorsum and hind paws of both male and female rats post dorsum incision, with evidence for sex dimorphism in the duration of primary hypersensitivity.

### 3.2 Morphine Attenuates Both Primary and Secondary Mechanical Hypersensitivity Following Dorsum Incision

Morphine (3 mg/kg) or saline (0.89% *w*/*v* NaCl) was administered 60 min prior to pain‐related behavioural testing on PSD 8 in experiment 2.

**FIGURE 8:**
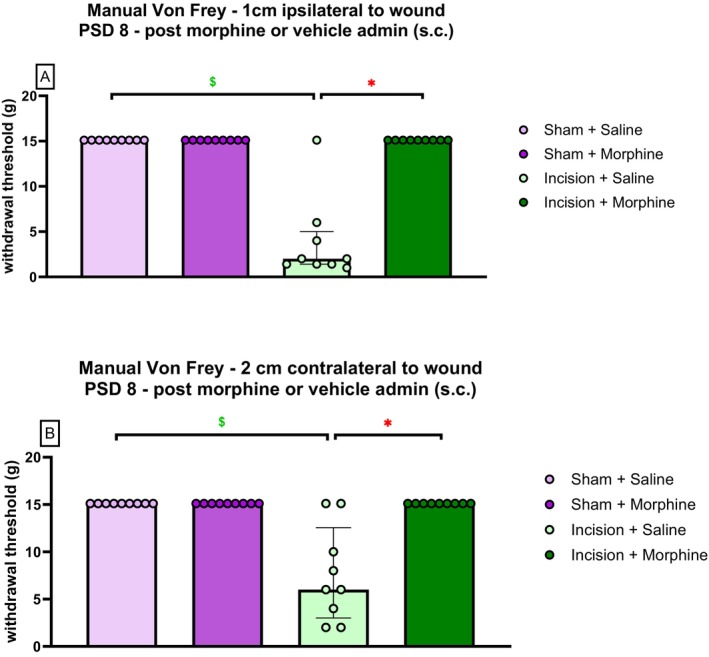
Effect of morphine (3 mg/kg s.c.) or saline administration (0.89% NaCl s.c.) on mechanical withdrawal thresholds at (A) 1 cm ipsilateral to the wound, and (B) 2 cm contralateral to the wound. Data are expressed as median ± interquartile range, *n* = 9 per group. Data were analysed using the Kruskal–Wallis test, followed by the Mann–Whitney *U* test with Bonferroni–Holm correction. * *p* < 0.01 Incision‐Saline versus Incision‐Morphine, $ *p* < 0.01 Incision‐Saline versus Sham‐Saline.

Kruskal–Wallis revealed significant differences in mechanical withdrawal threshold at 1 cm ipsilateral to the wound 60 min following morphine/saline administration on PSD 8: *χ* ^2^(3) = 29.42 (*p* < 0.01). Mann–Whitney *U* test with Bonferroni–Holm correction revealed a significantly lower mechanical withdrawal threshold at 1 cm ipsilateral to the wound in incision‐saline rats versus sham‐saline rats (*p* < 0.01) (Figure 8A). Mann–Whitney *U* test with Bonferroni–Holm correction revealed a significantly higher mechanical withdrawal threshold in incision‐morphine rats versus incision‐saline rats (*p* < 0.01), indicating that morphine (3 mg/kg s.c.) significantly attenuated incision‐related mechanical hypersensitivity at 1 cm ipsilateral to the wound (Figure 8A). There was no significant difference in the withdrawal threshold between the sham‐saline and sham‐morphine rats.

Kruskal–Wallis revealed significant differences in mechanical withdrawal threshold at 2 cm contralateral to the wound 60 min following morphine/saline administration on PSD 8: *χ* ^2^(3) = 24.98 (*p* < 0.01). Mann–Whitney *U* test with Bonferroni–Holm correction revealed a significantly lower mechanical withdrawal threshold at 2 cm contralateral to the wound in incision‐saline rats versus sham‐saline rats (*p* < 0.01) (Figure 8B). Mann–Whitney *U* test with Bonferroni–Holm correction revealed a significantly higher mechanical withdrawal threshold in incision‐morphine rats versus incision‐saline rats (*p* < 0.01), indicating that morphine (3 mg/kg s.c.) significantly attenuated incision‐related mechanical hypersensitivity at 2 cm contralateral to the wound (Figure 8B). There was no significant difference in withdrawal threshold between sham‐saline and sham‐morphine rats.

**FIGURE 9:**
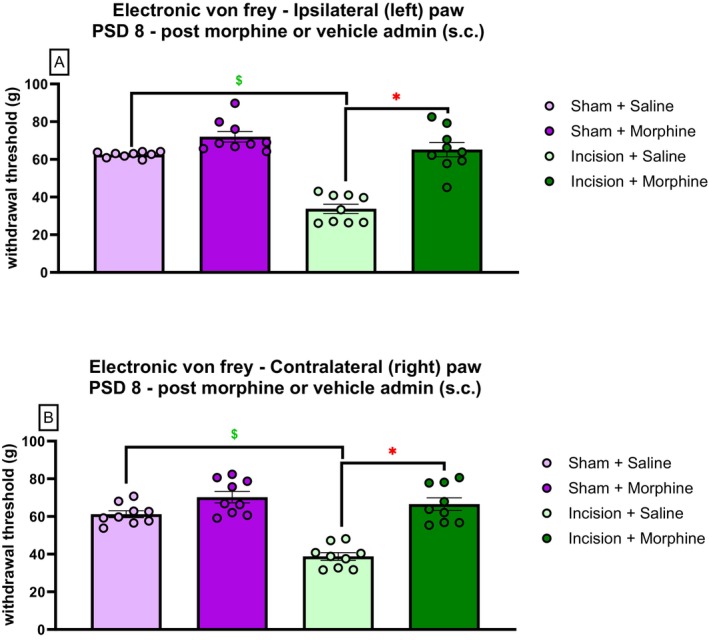
Effect of morphine (3 mg/kg s.c.) or saline administration (0.89% NaCl s.c.) on mechanical withdrawal thresholds following dorsum incision in the (A) ipsilateral (left) hind paw and (B) contralateral (right) hind paw. Data are expressed as mean ± S.E.M, *n* = 9 per group. Data were analysed using one‐way ANOVA, followed by Tukey's post hoc test. *p < 0.01 Incision‐Saline versus Incision‐Morphine, $*p* < 0.01 Incision‐Saline versus Sham‐Saline.

Two‐way ANOVA revealed a significant effect of procedure (F_(1,35)_ = 44.74, *p* < 0.001), drug (F_(1,35)_ = 58.82, *p* < 0.001) and drug × procedure (F_(1,35)_ = 17.08, *p* = 0.001) on ipsilateral paw withdrawal threshold at 60 min following morphine/saline administration on PSD 8. Tukey's post hoc test revealed a significantly lower ipsilateral paw withdrawal threshold in incision‐saline rats than in sham‐saline rats (*p* < 0.001). Tukey's post hoc test revealed a significantly higher ipsilateral paw withdrawal threshold in incision‐morphine rats than in incision‐saline rats (*p* < 0.001), indicating that morphine (3 mg/kg s.c.) significantly attenuated dorsum incision‐related mechanical hypersensitivity in the ipsilateral hind paw (Figure 9A). There was no significant difference in withdrawal threshold between sham‐saline and sham‐morphine rats.

Two‐way ANOVA revealed a significant effect of procedure (F_(1,35)_ = 24.41, *p* < 0.001), drug (F_(1,35)_ = 48.52, *p* < 0.001) and drug × procedure (F_(1,35)_ = 12.60, *p* = 0.001) on contralateral paw withdrawal threshold at 60 min following morphine/saline administration on PSD 8. Tukey's post hoc test revealed a significantly lower paw withdrawal threshold in the incision‐saline rats versus sham‐saline rats (*p* < 0.001). Tukey's post hoc test revealed significantly higher contralateral paw withdrawal threshold in incision‐morphine rats versus incision‐saline rats (*p* < 0.001), indicating that morphine (3 mg/kg s.c.) significantly attenuated dorsum incision‐related mechanical hypersensitivity in the contralateral hind paw (Figure 9B). There was no significant difference in withdrawal threshold between sham‐saline and sham‐morphine rats.

These results provide evidence that morphine attenuates both primary and secondary mechanical hypersensitivity following incision on the dorsum, providing evidence for the validity of the dorsum incision model as a model of pain.

## 4 Discussion

The results of these investigations indicate the development and persistence of both primary and secondary hypersensitivity following incision of the dorsum in rats of both sexes. Primary hypersensitivity at 1 cm ipsilateral to the wound persisted until Day 14 post‐incision in males and Day 7 in females. The presence of secondary mechanical hypersensitivity in the hind paws, persisting beyond the resolution of primary mechanical hypersensitivity, may be suggestive of central sensitisation following dorsum incision. Different approaches (manual von Frey filaments and electronic von Frey apparatus) were used to assess primary and secondary hypersensitivity respectively, and no direct comparison can or is being made between the two testing approaches. The effects described are based purely on the observed duration of hypersensitivity at distinct anatomical locations following dorsum incision, indicating differential time frames for primary and secondary hypersensitivity in this model. Administration of morphine (3 mg/kg) on post‐incision Day 8 significantly attenuated incision‐induced mechanical hypersensitivity in both the dorsum and hind paws of the incision rats, with no significant effect on mechanical withdrawal thresholds in the sham rats. The attenuation of mechanical hypersensitivity following morphine administration provides evidence for the predictive validity of the dorsum incision model as a model of incisional wound pain.

The development of mechanical hypersensitivity at 1 cm ipsilateral to the wound may be due to peripheral sensitisation involving primary afferent neurons. Peripheral sensitisation involves lowering the response thresholds to stimuli, an increase in receptive field size and an increase in spontaneous activity of primary afferent fibres (Brennan et al. 2005). Evidence suggests the innervation of the skin and subdermal muscles is unilateral, indicating that hypersensitivity observed at 2 cm contralateral to the wound cannot be due to injury of the primary afferents innervating this area (Theriault and Diamond 1988). Therefore, mechanical hypersensitivity at 2 cm contralateral to the wound may be due to alterations in pain pathways via other peripheral or central sensitisation mechanisms. Mechanical hypersensitivity at 2 cm contralateral to the wound may also be a form of mirror image pain; however, evidence for this phenomenon in the literature has focused on neuropathic pain (Jancalek 2011). Another potential mechanism underlying mechanical hypersensitivity at 2 cm contralateral could be an increase in receptive field size, following the incision and blunt dissection. Kawamata et al. (2005) have shown that surgical incision and suturing of hairy skin of the dorsum triggers enhanced wide‐dynamic range, high‐threshold and low‐threshold neuron excitability of the dorsal spinal cord. The authors concluded that the increased excitability of wide‐dynamic range neurons contributed to mechanical hypersensitivity following hairy skin injury in rats (Kawamata et al. 2005). The resolution of the 2 cm contralateral hypersensitivity from Day 4 post‐incision may indicate that receptive field size is returning to baseline beyond this time point, as mechanical hypersensitivity observed at 1 cm ipsilateral appears to peak 4 days post‐incision.

Mechanical hypersensitivity in the hind paws following dorsum incision indicates the presence of secondary hypersensitivity. Secondary hypersensitivity—an exaggerated response to stimuli applied to undamaged tissue distal to an injury—is common following tissue injury and inflammation (Van Den Broeke et al. 2016). Secondary hypersensitivity to punctate mechanical stimuli represents an enhanced response to input from non‐injured fibres. There is evidence for short‐lasting secondary hypersensitivity in the plantar incision model, indicating secondary hypersensitivity may be a common feature of incisional wound models (Zahn and Brennan 1999). Pogatzki et al. (2002) suggest that the mechanisms of secondary hypersensitivity following incision are inherent and unique to incision‐induced pain. These authors report that 40% of A‐delta and C fibres display spontaneous activity at post‐incision Day 1 following plantar incision (Pogatzki et al. 2002).

Duarte et al. (2005) investigated the effect of subcutaneous bupivacaine injection in the dorsum, at a site distant to the wound, 30 min prior to incision on post‐incision‐related pain. These authors report initial primary hyperalgesia is unaffected by subcutaneous bupivacaine prior to incision; however, the later pain‐related phenotype was attenuated. These authors hypothesise that these results indicate differential phases of post‐incisional wound‐related pain, an induction phase involving the sensitisation of peripheral nerves, and inflammation due to the wound, and a persistent phase, involving central sensitisation (Duarte et al. 2005). This contention is supported by the results of the investigations described herein, as hypersensitivity was observed in the hind paws beyond the resolution of the hypersensitivity in the dorsum, indicating central sensitisation.

A recent review by Mogil et al. (2024) highlights the sex differences in pain behaviour in various preclinical models. Sex differences and the importance of sex hormones in wound healing are well established. Castration of male mice accelerates cutaneous wound healing and reduces the inflammatory response (Ashcroft and Mills 2002). Ovariectomised young female rodents have delayed wound healing of acute incisional wounds, an effect reversed with the application of topical oestrogen. Topical oestrogen also promoted wound healing in non‐ovariectomised female rats (Ashcroft et al. 1997). The sex differences in the temporal profile of pain‐related behaviour following dorsum incisional wound creation described herein may be due to an increased wound healing and resolution ability in female rats compared to male counterparts. Further studies are required to test this hypothesis, with the specific aim of assessing wound healing in conjunction with pain‐related behavioural testing.

Morphine, when administered systemically, attenuates pain‐related behaviour in a variety of preclinical incision models (Flatters 2010; Martin et al. 2004; Zahn et al. 1997). Whiteside et al. (2004) report that morphine dose‐dependently attenuated mechanical hypersensitivity 24 h post‐paw incision, with maximum efficacy 1‐h following systemic administration (Whiteside et al. 2004). Following gastrocnemius incision, intrathecal morphine administration attenuated hind paw mechanical hypersensitivity, indicating that morphine is efficacious at reducing secondary hypersensitivity following incision on the hairy skin (Pogatzki et al. 2002). Previous research has shown that pain‐related behaviour 4 h following a 1 cm incision on the male rat dorsum is transiently reversed by morphine (2.5 mg/kg i.p.), 30 min following administration (Duarte et al. 2005). However, to our knowledge, the studies described herein are the first to investigate the effect of mu‐opioid receptor agonism at a later time point. The attenuation of mechanical hypersensitivity in the dorsum and hind paws by morphine 8 days post‐incision strongly indicates that the phenotype is pain‐related and enhances the validity of the dorsum incision model as a model for studying wound‐related pain. Morphine acts both peripherally and centrally to prevent the transduction of pain‐related signals to the supraspinal regions and to increase the activity of the descending inhibitory pain pathway (for review, see Bagley and Ingram (2020); Pathan and Williams (2012)), resulting in analgesia. The attenuation of both primary and secondary hypersensitivity following morphine administration is consistent with the known mechanism of action of morphine.

A limitation of the studies described herein is the use of male rats only to provide evidence for the predictive validity of the hairy skin back incision model as a model of pain in Experiment 2. We were unable to study the effects of morphine in both sexes due to animal licence‐related constraints stipulated on the permitted animal numbers to be included in each experimental group, coupled with the evidence for sex dimorphism in the time course of pain‐related behavioural phenotype development post‐incision in Experiment 1 which was not conducive to using groups of mixed sex. Therefore, the choice to proceed with males for Experiment 2 was made based on the evidence for a more robust pain phenotype in males post‐incision, with primary hypersensitivity persisting to post‐incision Day 14 in males and Day 7 in females in Experiment 1. However, future research using female rodents is imperative to ensure the maximum translational relevance of this model and to pharmacologically validate the pain‐related behavioural phenotype observed in female rats following incision. As highlighted in a recent review by Segelcke et al. (2025), many studies in the area of post‐incision or post‐surgical pain are limited by their use of young, male rodents—and use of evoked mechanical readouts. With this consideration, another limitation of the studies described herein was the use of mechanical measures only to measure pain‐related behaviour. Testing for potential cold hypersensitivity at the dorsum and hind paws was carried out in a pilot feasibility study that provided no evidence to suggest altered response to cold (data not shown). Logistically, due to the location of the wound on the dorsum of the rat, it is not feasible to carry out testing for other modalities such as heat hypersensitivity without custom built equipment. The purpose of these studies was to characterise and validate the model of wound‐related pain in the context of evoked mechanical hypersensitivity; however, we recognise that the included data are non‐exhaustive to the potential utility of the model. Further studies should endeavour to include additional testing across multiple domains, including measures of spontaneous, ongoing, or non‐evoked pain for further characterisation as appropriate.

Overall, these results extend the behavioural characterisation of the rat dorsum incision model, demonstrating for the first time that the model is associated with secondary hypersensitivity in the anatomically distinct location of the hind paws. These results provide, to our knowledge, the first comparison between males and females for pain‐related behaviour in the dorsum incision model, providing evidence for sex dimorphism in pain‐related behaviour following dorsum incision. Furthermore, the data provide evidence to pharmacologically validate the pain‐related behavioural phenotype observed post‐incision, strongly indicating that the phenotype observed post‐incision is pain‐related. The results support the use of the dorsum incisional wound model for preclinical study of wound‐related pain and related novel therapeutics.

## Author Contributions

This study was designed by C.R.H., M.C.R. and D.P.F. The experiments were performed by C.R.H., M.C.R. and M.H. The data were analysed by C.R.H., and the results were critically examined by all authors. C.R.H. had a primary role in preparing the manuscript, which was edited first by D.P.F., and then by all other authors. All authors reviewed and approved the final version of the manuscript and agree to be accountable for all aspects of the work.

## Funding

Funding was provided by the Research Ireland—Taighde Éireann Government of Ireland Postgraduate Scholarship Programme (GOIPG/2021/1434, GOIPG/2020/1496 and GOIPG/2023/2741), and by B. Braun Hospicare and Research Ireland—Taighde Éireann with co‐funding under the European Regional Development Fund under Grant 13/RC/2073‐P2.

## Ethics Statement

The experimental procedures were approved by the Animal Care and Research Ethics Committee of University of Galway. These experiments were completed under a licence from the Health Products Regulatory Authority in the Republic of Ireland, in accordance with the EU Directive 2010/63. These studies were designed, conducted and reported in accordance with the ARRIVE 2.0 guidelines.

## Conflicts of Interest

The authors declare no conflicts of interest.
